# Associations of perceived social support, resilience and posttraumatic growth among young and middle-aged patients with first-episode psychosis

**DOI:** 10.3389/fpsyt.2025.1538275

**Published:** 2025-04-29

**Authors:** Chongzheng Zhao, Xiumei Zhao, Weiyu Teng, Guiyuan Zou

**Affiliations:** ^1^ Faculty of Arts and Social Sciences, The University of Sydney, Sydney, NSW, Australia; ^2^ Shandong Mental Health Center, Shandong University, Jinan, Shandong, China

**Keywords:** resilience, perceived social support, posttraumatic growth, first-episode psychosis, mediation

## Abstract

**Background:**

The prevalence of mental disorders among young and middle-aged populations has demonstrated a significant upward trend, with first-episode psychosis (FEP) frequently associated with psychological distress and functional impairments during initial onset. While persons affected by FEP frequently report psychological distress and reduced quality of life during early illness stages, they may also experience post-traumatic growth (PTG), which fosters positive changes that facilitate their recovery. Yet there is limited attention on PTG in young and middle-aged patients with FEP. Therefore, this study aimed to investigate the level of PTG and identify significant correlates and mediators of PTG among young and middle-aged patients with FEP.

**Methods:**

From January 2021 to December 2023, two hundred eight patients with first-episode psychosis were enrolled from a tertiary hospital in Shandong Province, China, through convenience sampling. The Perceived Social Support Scale (PSSS), Posttraumatic Growth Inventory-Short Form (PTGI-SF), and Connor-Davidson Resilience Scale (CD-RISC10) were administered. Hierarchical linear regression modeling was performed to examine the associations between perceived social support and PTG and the mediating effect of resilience.

**Results:**

The PTG score was 31.22 ± 6.59, and resilience and PSS could positively predict the variance in PTG. Resilience partially mediated the relationship between PSS and PTG, and the value of the mediating effect was 22.8%.

**Conclusions:**

Young and middle-aged patients with FEP have a moderate level of PTG. Resilience partially mediates the relationship between PSS and PTG. Therefore, interventions focusing on promoting PSS and resilience should be developed to encourage PTG in young and middle-aged patients with FEP.

## Introduction

Young and middle-aged adults are the primary demographic responsible for fulfilling family obligations, advancing careers, and contributing to society. This demographic confronts a complex array of stressors spanning professional competition, financial instability, parenting pressure, and interpersonal tensions (1, 2). The interaction between these stressors and genetics and biology may trigger or exacerbate the onset of psychosis (3). Notably, the onset age of most psychosis (such as schizophrenia and mood disorder) is concentrated in young and middle-aged adults. A diagnosis of psychosis at this stage is a profoundly threatening and traumatic event that severely impacts occupational performance, family cohesion, social participation and quality of life, ultimately leading to a substantial socioeconomic burden (4, 5). Psychological adjustment following a diagnosis of psychosis is not uniformly related to negative psychological consequences (e.g., posttraumatic stress disorder, burden and depression) but is related to posttraumatic growth (PTG), a positive outcome in the aftermath of psychotic episodes (6, 7). Moderate increases in PTG have been reported among patients with FEP (8). However, the situation and mechanism of PTG among young and middle-aged patients with FEP remains unknown. This study aimed to investigate PTG and its relationship with perceived social support and resilience among young and middle-aged patients with FEP.

PTG is usually described as a positive transformation after an individual experiences a traumatic event (9). The emotional-cognitive processing model of PTG explains the generation process of PTG: Individuals who have experienced a traumatic event will initiate cognitive processing, assess the nature of the trauma, and adopt various emotional responses and coping strategies. These emotional responses and coping strategies subsequently influence cognitive processing, ultimately facilitating positive psychological transformations through adaptation or assimilation (9, 10). Research on PTG has examined people with accidental trauma (11), natural disasters (12) and chronic diseases such as cancer (13); relevant research on young and middle-aged patients with FEP is limited. PTG following FEP reported consistent changes in PTG, such as new perception, positive changes in developing positive character traits, making positive lifestyle changes, developing stronger connections with others, integrating the FEP with the self, experiencing greater religiosity and appreciating life (6–8). However, PTG does not occur naturally (7, 8). Studies have identified predictors of PTG, including direct precipitating factors (trauma exposure severity) (9, 10), individual stable factors (socio-demographic characteristics) (14, 15), environmental factors (social support) (16), and state-related factors (rumination, resilience, coping strategies) (17, 18), all contributing directly or indirectly to PTG.

Perceived social support (PSS) is frequently defined as an individual’s perception of the availability of support from others (19). The emotional-cognitive processing model posits that the processing speed and depth of the emotion-cognition cycle, which ultimately lead to PTG, depend on the social support system (9, 11). PSS not only serves as a pivotal factor in advancing rehabilitation, stabilizing symptoms, and facilitating functional recovery among patients with psychosis (20, 21), but also Sarason further highlights that PSS promote PTG through enhanced positive self-imagination, self-acceptance, hope, love, and satisfaction (22). Moreover, many studies have indicated that individuals with FEP exhibit a significantly reduced capacity to secure social support compared to both the general population and other chronic disease groups (23, 24). However, few studies to date have assessed the effect of PSS on PTG among young and middle-aged patients with FEP.

Resilience, increasingly recognized as a dynamic internal resource or process, reflects an individual’s ability to positively react, adapt, and grow through adversities and stressors (25, 26). Even within psychiatric populations, higher resilience were significantly associated with lower severity of specific symptomatology (positive, negative, depressive symptoms, suicidal ideation, cognitive deficits) and better insight, as well as favorable illness course indicators, adaptive internal factors (reduced stigma, enhanced self-esteem), and superior psychosocial function encompassing real-life, social and interpersonal functioning, and quality of life (26–29). Individuals with high resilience demonstrate enhanced PTG through adaptive cognitive processing of adversities (28, 30). Bensimon reported that resilience is one of the promoting factors for PTG (31). Currently, several studies have detected the correlation among resilience, perceived social support and PTG in different contexts (30, 32, 33), and social support may promote resilience in response to major adversity (34, 35). There is still a lack of significant correlates and mediators of PTG among young and middle-aged patients with FEP. We therefore speculated that PSS may affect PTG of young and middle-aged patients with FEP through resilience.

Research has underscored the great significance of studying PTG and its intervention strategies (36). PTG’s significance in psychosis patients lies not only in its capacity to reflect psychological well-being through enhanced personal recovery and quality of life, but also in its ability to enable deeper contemplation of traumatic experiences and the adoption of proactive coping mechanisms (4, 37, 38). Transforming the negative impacts of psychotic experience into positive ones and promoting PTG are therefore important. This study explored the mediating effect of resilience between PSS and PTG among young and middle-aged patients with FEP to afford a theoretical basis for intervention measures formulation.

## Methods

### Study design and participants

This study was approved by the Ethics Committee of Shandong Mental Health Center. From January 2021 to December 2023, we conducted a cross-sectional survey of patients with young and middle-aged FEP from a tertiary hospital in Shandong Province, China, through convenience sampling. Given the tendency of individuals with psychosis to conceal their symptoms due to stigma, which significantly reduces motivation for research participation, the convenience sampling methodologies allows researchers to more effectively access participants that are willing to disclose their diagnostic status during recruitment phases. In this survey, the inclusion criteria included the following: (1) were native Chinese speakers; (2) had been diagnosed with psychosis by the International Statistical Classification of Diseases, Tenth Revision (ICD-10); (3) were experiencing a first episode of psychosis; (4) were aged between 18 and 59 years at the time of hospitalization; and (5) were in a stable state, with no difficulty in communication or cooperation. The exclusion criteria were as follows: (1) patients with severe physical diseases, organic brain lesions or other serious medical conditions and (2) patients with intellectual disabilities who could not communicate normally. All participants and their legal guardians, having been fully informed of the study’s objectives and their right to withdraw at any time, provided written informed consent prior to participation, with assurances that all data would remain anonymized, securely stored, and exclusively utilized for this research.

### Procedure

Sampling and data collection were performed through face–to-face interviews. First, the principal investigator contacted the head nurse of each ward and invited them to act as supervisors and facilitators of the study. They are responsible for screening participants who meet the inclusion and exclusion criteria. Second, once qualified participants were confirmed, they were informed orally and in writing of the study’s purpose, along with information on their rights as participants and the anonymity of this research project; we invited them to participate in our survey (i.e., a self-administered hard-copy questionnaire), which they were instructed to complete in a staff room or health education room following their daily treatment. For those unable to complete the questionnaire independently, the researcher provided assistance by reading the items and recording the answers objectively. All questionnaires required 15–20 minutes to complete, and the researcher reviewed the questionnaire immediately after completion and collected it on the spot after asking the participants to add any missing items. Finally, the questionnaire was distributed to 224 invited participants, and 208 questionnaires were successfully completed, resulting in a response rate of 92.86%.

### Instruments

#### Perceived Social Support Scale

The PSS was measured via the Chinese version of the Multidimensional Scale of Perceived Social Support (MSPSS); 12 items evaluate three types of support: family, friends, and significant others (19, 39). The responses used a 7-point Likert scale (1 = *very strongly disagree*, 7 = *very strongly agree*). In the present study, Cronbach’s α was 0.88, 0.92, and 0.87 for support from family, friends, and significant others, respectively.

#### Connor-Davidson Resilience Scale

The self-reported CD-RISC10 originates from the Connor–Davidson Resilience Scale and measures resilience via 10 items. The responses used a 5-point Likert scale (0 *= not at all*, 4 *= nearly all the time*) (40). The total scores on the CD-RISC-10 range from 0–40; higher scores indicate greater resilience. The CD-RISC-10 has shown excellent internal consistency and construct validity. The reliability and validity of the CD-RISC10 have been supported in Chinese samples (41). In this study, the scale’s Cronbach’s α was 0.90.

#### Posttraumatic Growth Inventory-Short Form (PTGI-SF)

The self-reported PTGI-SF originates from the Posttraumatic Growth Inventory (9) and measures favorable outcomes after a traumatic event via 10 items (42), including 5 dimensions: relating to others, new possibilities, personal strength, spiritual change and appreciation of life. The responses used a 6-point scale (0 = not at all, 5 =to a very great degree); higher scores indicated greater PTG. The internal consistency of the total scale was acceptable. In this study, the scale’s Cronbach’s α was 0.91.

### General information of the participants

The participants were asked to provide their general information, including their age, sex, years of education, marital status, perception of family economic status and region of residence.

### Statistical analysis

Statistical analysis was performed with SPSS v.24.0. Descriptive statistics for PTG, resilience, the PSS, and general information were calculated. *T* tests or ANOVA were used to evaluate differences in participants’ PTG. Pearson’s *r* was calculated to examine the correlations among PTG, resilience, and the PSS among young and middle-aged patients with FEP. According to the pioneering work in mediation models by Baron and Kenny (43), a hierarchical regression model was estimated to examine the mediating role of resilience between PSS and PTG. The following requirements for such analysis were verified: a significant correlation between PSS (independent variable) and PTG (dependent variable); a significant correlation between PSS and resilience (the mediator) and between resilience and job burnout. Additionally, the effect of PSS on PTG should shrink (partial mediator) or become statistically insignificant (full mediator) after the inclusion of resilience in the model. Standardized estimates (*β*), F statistics, determination coefficient (R^2^), and R^2^ -changes (ΔR^2^) for each step were provided. Finally, the mediating effect of resilience between PSS and PTG was examined using model 4 of PROCESS v.3.5 (44). The significance level was set at 2-sided p < 0.05.

## Results

### General information of the participants

The participants’ demographic and clinical characteristics are presented in **Table 1**. The participants’ mean age was 44.38 ± 10.13 years; more than two-thirds were male (67.3%). Approximately half of the participants were married. In terms of years of education, 31.3% had been educated for less than 6 years, 38.9% had been educated for 6–9 years, 21.2% had been educated for 9–12 years, and 8.7% had been educated for more than 12 years. For poor economic conditions, balance and higher accounted for 23.1%, 62.5% and 14.4%, respectively. For the region of residence, 59.1% were living in rural areas.

**Table 1:** General information of participants distribution of posttraumatic growth in categorical items.

Variable	N (%)	Post-traumatic growth (Mean ± SD)	t/F	P
**Age group**			4.332	0.039
18~44 years old	98(47.1)	32.45±5.84		
45~59 years old	110(52.9)	30.12±7.03		
**Gender**			9.326	0.003
Male	140(67.3)	31.75±6.03		
Female	68(32.7)	30.12±7.54		
**Marital status**			6.511	0.011
Married	109(52.4)	33.92±5.30		
Single	99(47.6)	28.24±6.61		
**Years of education**			29.829	<0.001
≤ 6 years	65(31.3)	26.22±6.19		
6 ~ 9 years	81(38.9)	32.30±5.43		
9-12 years	44(21.2)	34.20±4.81		
>12 years	18(8.7)	37.11±5.10		
**Economic conditions**			13.582	<0.001
Poor	48(23.1)	27.27±6.71		
Balance	130(62.5)	32.06±6.18		
Higher	30(14.4)	33.87±5.54		
**Region of Living**			0.023	0.879
Rural	123(59.1)	30.26±6.37		
Urban	85(40.9)	32.60±6.69		

### Means and correlations between PTG, perceived social support, and resilience

The average PTG score was 31.22 ± 6.59, the average resilience score was 29.46 ± 6.79, and the score for perceived social support was 31.22 ± 6.59. was 62.96 ± 9.65 (**Table 2**). PTG scores varied significantly depending on age group, gender, marital status, years of education, and economic conditions but not depending on the region of residence (**Table 1**).

**Table 2:** Mean, standard deviations (SD) and correlation of variables.

Variables	Mean ± SD	1	2	3
1 Perceived social support	62.96 ± 9.65	1		
2 Resilience	29.46 ± 6.79	0.285^**^	1	
3 Posttraumatic growth	31.22 ± 6.59	0.298^**^	0.468^**^	1

**P < 0.01.

**Table 2** shows the correlations between the study variables. PTG was positively related to resilience and the PSS (*r* = 0.468, *p* < 0.001; *r* = 0.298, *p* < 0.001). The relationship between resilience and PSS scores was significantly positive (*r* = 0.285, *p* < 0.001).

### Mediation of resilience between PSS and PTG

Considering the significant association between PSS scores and PTG, we examined whether resilience mediated this association, the results of which are presented in **Table 3**. In blocks 1–3, the PSS, resilience and general information of the participants explained 46.3% of the variance in PTG. The control variables explained a further 39.5% of the variance in PTG in Step 1. After controlling for the general information of the participants in Block 1, the regression analysis in Step 2 indicated that the relationships between PSS (*β* = 0.208, *P* < 0.001) and PTG were positive and significant, explaining 4% of the variance in PTG. Resilience was added in Step 3 and was found to be significantly and positively correlated with PTG (*β* = 0.200, *P* < 0.001), explaining a further 2.8% of the variation in PTG. PSS was significantly and positively associated with PTG (*β* = 0.160, *p* < 0.01).

**Table 3:** Hierarchical linear regression analysis results.

Variable	Post-traumatic growth		
	Step 1 (β)	Step 2 (β)	Step 3 (β)
Block 1			
Age	-0.102	-0.112*	-0.080
Gender	0.025	0.027	0.017
Marital status	0.308***	0.282***	0.256***
Years of education	0.400***	0.386***	0.324***
Economic conditions	0.158**	0.148**	0.145**
Block 2			
Perceived social support		0.208***	0.160**
Block 3			
Resilience			0.200**
*F*	28.017***	27.563***	26.535***
*R^2^*	0.410	0.451	0.482
*△R^2^*	0.395	0.435	0.463

***P < 0.001 **P < 0.01.

After adding resilience to the regression model, the *β* coefficient of the PSS decreased (from 0.208 to 0.160, *P* < 0.01), indicating that resilience partially mediated the association of the PSS with PTG. The results of the bootstrapping method (**Table 4**) revealed that the path coefficient of the indirect effect of PSS on PTG was 0.0324 (95% CI: 0.0155, 0.0894), supporting the assertion that resilience is a partial mediator. The indirect effect of PSS on PTG through resilience accounted for 22.8% of the total effect.

**Table 4:** Resilience mediates the relationship between perceived social support and Post-traumatic growth.

Mediation model hypothesis	Effect	Point estimate	SE	95% CI
				LL UL
Perceived social support -Resilience -Post-traumatic growth	Total effect	0.1418	0.0362	0.0704 0.2133
	Direct effect	0.1095	0.0365	0.0374 0.1815
	Indirect effect	0.0324	0.0128	0.0155 0.0894

## Discussions

This study first investigated the correlates of PTG and explored the relationships among PSSs, resilience, and PTG among young and middle-aged patients with FEP. This study is the first to focus on the mediation of resilience between PSS and PTG. Specifically, the PTG scores were above the medium level; the PTG scores varied significantly depending on age group, gender, marital status, years of education, and economic conditions but not depending on the region of residence; PSS and resilience were significant positive predictors of PTG; and resilience partially mediated the relationship between PSS scores and PTG among young and middle-aged patients with FEP.

The total PTG scores in this study resembled previously reported values (8, 45). However, evidence of PTG in persons with FEP is insufficient. The underlying cause of this situation may be attributed to societal prejudices surrounding psychosis. Furthermore, some medical professionals believe that it is challenging for most patients to achieve complete functional recovery, let alone to experience positive changes (46). Age was negatively associated with PTG, which was inconsistent with existing research results (15, 47, 48). Current research identifies age-dependent divergence in PTG mechanisms: younger individuals, unburdened by family or professional roles, exhibit attenuated stigma that fosters PTG through intentional self-exploration (15), whereas older groups, constrained by role-specific stressors (e.g., career progression, intergenerational caregiving), often prioritize illness concealment to preserve social equilibrium, consequently limiting PTG opportunities (48). Surprisingly, our study revealed that male patients had significantly higher PTG scores than female patients, which disagreed with previous findings (47, 49). This observed divergence may stem from sociostructural norms and gender role expectations. While males are often expected to demonstrate resilience, independence, and problem-solving skills that may facilitate PTG, females are generally more permitted to express vulnerability through emotionally-focused coping styles rather than active problem-solving approaches, potentially attenuating PTG translation efficiency (10, 49, 50). Patients who reported a higher level of education exhibited greater levels of PTG, potentially mediated through education-enhanced cognitive accuracy and adaptive psychological processing of psychosis (51, 52). Moreover, patients demonstrating higher educational attainment with higher levels of education are more likely to adopt optimistic attributions and generate more PTG (53, 54). These findings suggest that higher educational attainment—a proxy for cognitive reserve—may confer neuroprotective benefits (55, 56), as evidenced by attenuated cognitive decline in individuals with psychosis. Specifically, education may enhance compensatory mechanisms that maintain performance in executive function, working memory, and processing speed despite neuropathological burden (57). Patients with high incomes tend to experience less anxiety regarding economic issues. Consequently, they are more inclined to invest their willingness, energy, and opportunities in actively engaging with treatment, thereby facilitating the development of PTG (53). Marriage and PTG are positively correlated, which may be because married patients in disease states receive more social support (58).

Our findings indicate that PTG was positively correlated with PSS among young and middle-aged patients with FEP, which is consistent with the findings of Jordan et al. (59). These finding supports the social support buffer hypothesis, indicating that PSS serves as a protective factor for individuals facing major life challenges including psychosis by facilitating mindset adaptation, effectively manage their mentality, resulting in promotion of PTG (60). Meanwhile, PSS can transform trauma experiences into growth by activating and promoting an individual’s emotional-cognitive cycle (9, 10). Moreover, Robust PSS may benefit young and middle-aged patients with FEP in fostering PTG by creating a safe and comfortable living atmosphere that enables them to reappraise traumatic events from a positive perspective (61). Specifically, family support, particularly spousal understanding and affection, plays a pivotal role in the psychosocial adaptation of psychosis patients by mitigating anxiety and perceived inferiority, while fostering a sense of dignity and care that empowers proactive life orientation and enhanced illness self-management (62, 63). Support from friends, colleagues, peers and healthcare providers significantly facilitates PTG in individuals with mental illnesses through multidimensional mechanisms: workplace social integration (colleagues) (64), shared experiential validation (peers) (65), emotional scaffolding (friends) (66, 67), and clinical empowerment (healthcare providers) (68), collectively fostering cognitive reframing, self-efficacy enhancement, and meaning reconstruction post-trauma. Third, highlighting the significance of community medical institutions and nonprofit organizations focused on psychosis is vital (68). Therefore, a well-established support network consisting of family, friends, colleagues, peers, healthcare providers and community medical institutions and nonprofit organizations should be recommended as best evidence for promoting PTG in young and middle-aged patients with FEP.

Resilience research has empirically validated its role as an endogenous protective mechanism against trauma-induced suffering and quality-of-life deterioration, with empirical evidence (69) demonstrating robust correlations between elevated resilience and superior psychological outcomes. These may be because high-resilience individuals have more effective reappraisal and problem-focused coping strategies and are therefore able to find positive meaning in their trauma (70, 71). The cognitive processing of a diagnosis (particularly rumination) critically also affects positive outcomes (17). Soo et al. reported that intrusion and instrumental rumination are positively associated with PTG and that high-resiliency individuals typically experience greater intrusive rumination, which in turn is related to greater PTG (72, 73). Simultaneously, resilience was directly associated with greater challenge appraisal, which was then associated with higher levels of PTG (73). Resilience may therefore importantly determine PTG. These findings suggest that interventions designed to enhance resilience may facilitate the development of PTG among young and middle-aged patients experiencing FEP. Consequently, targeted interventions may become necessary for promoting PTG.

This study is especially important because it is the first empirical attempt to reveal how resilience mediates the relationship between PSS and PTG among young and middle-aged patients with FEP. These results enrich previous findings about the explanatory mechanisms underlying the PSS-PTG relationship. High-resiliency patients with FEP can show positive emotions such as optimism, tenacity and self-improvement when suffering from psychotic disorders and take actions such as actively seeking external support to promote physical and mental health and relieve negative emotions. Conversely, low-resiliency patients with FEP are prone to escape behaviors such as social withdrawal and avoidance of social activities in the face of stressful events, so they receive less social support and are more likely to experience negative emotions such as pessimism, despair, and shame. Therefore, it is particularly important for medical staff to strengthen the psychological resilience of patients when they intervene in the PTG of mental illness. Clinical attention should be given to the psychological status of patients to help patients establish a tough, optimistic and self-improving mentality and reduce the generation of negative psychological problems such as stigma.

The first limitation of our study include its cross-sectional design, which restricts our ability to draw causal inferences. Secondly, convenience sampling may predominantly recruit patients actively seeking clinical care, potentially excluding those with mild symptoms or refuse treatment. This exclusion introduces selection bias, resulting in sample representative bias. Meanwhile, patients who volunteer to take part in the study could possess specific traits (for instance, a higher educational level, a stronger drive for recovery), which result in volunteer bias. Thirdly, the sample size was relatively small and restricted to young and middle-aged patients with FEP from a single a single hospital, which may have limited the statistical power and restricted the generalizability of our results. Finally, individual cognitive style and psychotic symptoms might also be influencing factors for PTG. We did not address this in our study. Future research should therefore further examine the associations between individual cognitive style, psychotic symptoms and PTG.

## Conclusion and practical implications

Patients with FEP exhibited moderate PTG, with PSS and resilience influencing PTG. This study identified resilience as a partial mediator between PSS and PTG, highlighting the potential for targeted interventions to amplify adaptive coping mechanisms among young and middle-aged patients with FEP. Therefore, it is recommended that medical staff implement resilience interventions, such as mindfulness training, and establish social support systems that foster collaboration among hospitals, communities, and families to increase PTG in young and middle-aged patients with FEP. Specifically, healthcare professionals should seek opportunities to collaborate with psychotherapists to provide resilience training and education to patients regularly, including mindfulness interventions, cognitive behavioral therapy, and psychological health courses. Therefore, the government should integrate Resilience-Building Program into the national mental health agenda and prioritize funding for Resilience-Building Program in public mental health services, with a focus on FEP early intervention clinics. Additionally, nurses should encourage patients with FEP to actively pursue available social support resources, such as support from family members, spouses, friends, and neighbors, to bolster their resilience. Patient clubs and peer support groups organized by healthcare providers may also assist patients in obtaining emotional and informational support. Hence, empowering cross-sector collaboration protocols between healthcare systems and community organizations to institutionalize hospital-community-family support networks is also a necessary initiative. Finally, emerging evidence highlights cross-cultural divergences in PTG manifestations, with collectivist societies demonstrating heightened relational growth (e.g., familial reconciliation) versus individualist cultures’ emphasis on self-actualization growth. Thus, cross-culturally responsive PTG protocols for FEP populations with increased transnational mobility patterns represent a necessary advancement in global mental health implementation frameworks.

## Data Availability

The raw data supporting the conclusions of this article will be made available by the authors, without undue reservation.
